# Vaginal colonisation of women in labour with potentially pathogenic bacteria: a cross sectional study at three primary health care facilities in Central Uganda

**DOI:** 10.1186/s12879-020-4821-6

**Published:** 2020-01-31

**Authors:** Josephine Tumuhamye, Hans Steinsland, James K. Tumwine, Olive Namugga, David Mukunya, Freddie Bwanga, Halvor Sommerfelt, Victoria Nankabirwa

**Affiliations:** 1 Centre for Intervention Science in Maternal and Child Health (CISMAC), Centre for International Health, Department of Global Public Health and Primary Care, University of Bergen, Bergen, Norway; 2 CISMAC, Centre for International Health, Department of Global Public Health and Primary Care and Department of Biomedicine, University of Bergen, Bergen, Norway; 3 Department of Paediatric and Child Health, Makerere University, Kampala, Uganda; 4 CISMAC, Centre for International Health, Department of Global Public Health and Primary Care, University of Bergen, Bergen, Norway; 5 Department of Immunology and Molecular Biology, Makerere University, Kampala, Uganda; 6 Department of Epidemiology and Biostatics school of Public Health, Makerere University, Kampala, Uganda

**Keywords:** Potentially pathogenic bacteria, Vaginal colonisation, Labour, *Escherichia coli*, *Klebsiella pneumoniae*, *Staphylococcus aureus*, Uganda

## Abstract

**Background:**

Potentially pathogenic bacteria that colonise the lower genital tract of women in labour can be passed to the baby during birth. While many babies become colonised with these bacteria after delivery, a few develop neonatal infections. The lower genital tract is a reservoir for potential pathogens and a source of infection for neonates. We determined the prevalence of vaginal colonisation of potentially pathogenic bacteria among women in labour in Central Uganda and identified potential risk factors associated with this colonisation.

**Methods:**

We conducted a cross sectional study at three primary health care facilities and collected vaginal swabs from HIV-1 negative women in labour. Specimens were cultured on different selective microbiological media, and biochemical tests were used to classify bacterial isolates on the species level. Multivariable logistic regression analyses were used to estimate the association between relevant exposures and colonisation with potentially pathogenic bacteria.

**Results:**

We recruited 1472 women in labour whose mean age was 24.6 years (standard deviation [SD] 4.9). Of these, 955 (64.9%; 95% Confidence Interval [CI] 62.4, 67%) were vaginally colonised with at least one potentially pathogenic bacterial species. The most commonly isolated species were *Escherichia coli* (*n* = 508; 34.5%), *Klebsiella pneumoniae* (*n* = 144; 9.8%) and *Staphylococcus aureus* (*n* = 121; 8.2%). Results from exploratory multivariable regression analyses indicated that having had ≥5 previous pregnancies (adjusted odds ratio [aOR] 0.59; 95% CI 0.35, 0.97) or being ≥30 years old (aOR 1.52; 95% CI 1.03, 2.23) could be associated with vaginal colonisation with any potentially pathogenic bacteria, as well as with vaginal colonisation with *S. aureus* (aOR 0.33; 95% CI 0.12, 0.88, and aOR 2.17; 95% CI 1.17, 4.00, respectively). Possession of domestic animals in a household (aOR 0.57; 95% CI 0.35, 0.92) could be associated with vaginal colonisation with *E. coli.*

**Conclusions:**

Two-thirds of HIV-1 negative women in labour were vaginally colonised by potentially pathogenic bacteria, mainly *E. coli, K. pneumoniae*, and *S. aureus*.

## Background

The normal lower genital tract is inhabited by a number of different bacteria that live in well-balanced populations. In healthy women of reproductive age, the primary bacteria colonising the vagina are of the genus *Lactobacillus* [1]. They reduce the pH of the vagina to between 2 and 4, which helps to inhibit growth of pathogenic bacteria [2]. During pregnancy, physiological changes alter the homeostasis of the vaginal environment. These changes are complex and not fully understood, but they generally lead to a reduction in the *Lactobacillus* population and, thereby, facilitating the growth of potentially pathogenic bacteria such as *Staphylococcus aureus* and members of the *Enterobacteriaceae* family [3].

During vaginal delivery, a newborn comes into direct contact with the mother’s flora in the lower genital tract. Eventually, the baby’s umbilicus, mucous membranes and parts of the skin may be colonised with bacteria that are potentially pathogenic for the neonate [4]. This colonisation process is known as seeding, and it has implications for long term neonatal health outcomes [5]. The potentially pathogenic bacteria that are seeded to the baby often include *S. aureus,* group B *Streptococcus* (GBS)*,* group A *Streptococcus* (GAS), *Enterococcus* spp., *Klebsiella pneumoniae, Escherichia coli, Enterobacter* spp., *Pseudomonas* spp*.* and *Citrobacter* spp. [6].

Severe infections account for 26% of neonatal deaths globally [7] and they are the leading cause of mortality among newborns in sub Saharan Africa [8]. The lower genital tract of women is an important source of pathogens causing life-threatening infections including bacteremia, meningitis, pneumonia and arthritis during the first week of life [9, 10] [11]. We conducted a cross-sectional study among women in labour at three primary health care facilities in the Central region of Uganda to estimate the prevalence and improve our understanding of the aetiology and risk factors associated with vaginal colonisation by potentially pathogenic bacteria.

## Methods

### Study design and setting

This cross-sectional study was conducted between July 2016 and July 2018 in three primary health care facilities in Central Uganda: Mukono Health Centre IV, Kawaala Health Centre III, and Kitebi Health Centre III. These three health care facilities have a combined monthly average of 2400 antenatal visits and 1200 deliveries. The three health facilities mostly deliver women who are considered to have low risk of obstetric complications. The women who are likely to have complicated deliveries are usually referred to tertiary hospitals. The HIV prevalence among women in reproductive age in Uganda is approximately 8% [12]. The Mukono Health Centre IV is located within Mukono district, which has a largely rural population of around 60,000 people and is located around 25 km from Uganda’s capital city, Kampala, while the Kitebi Health Centre III and the Kawaala Health Centre III are located in Kampala, which has a population of approximately 1.5 million people. The study was nested within an ongoing randomised controlled trial aimed at assessing the effectiveness of a single application of 4% chlorhexidine solution on the umbilical cord stump for the prevention of omphalitis and severe illness in HIV-1 unexposed newborns [13].

### Participants

We included women who became enrolled in the above-mentioned randomised controlled trial, who were HIV-1 negative, who gave birth during the daytime on a weekday, who gave consent to participate in the study before (orally) and within 12 h (written) after giving birth, and who gave birth to babies who had the following characteristics: birth weights of > 1.5 kg, no severe congenital anomalies, no obvious signs of cord stump infection, and no severe illness requiring hospitalisation at birth [13]. The randomised controlled trial aims to recruit 4760 newborns, and we enrolled 1472 of these into the present study. With this sample size we would obtain a very high (0.7 to 2.6%) absolute precision, i.e. the difference between the upper limit and the lower limit of the 95% confidence interval (CI) for prevalence values ranging from 2 to 50%. Demographic characteristics of the study participants were collected through interviews, as described below, and can be found listed in Table 1.

**Table 1:** Distribution of characteristics of study participants at the three study sites

Participant characteristics	*N* = 1472 (%)	Uncolonised 517 (%)	Colonised 955 (%)
Mother’s age			
< =19 years	205 (13.9)	76 (14.70)	129 (13.5)
20–24 years	587 (39.9)	216 (41.8)	371 (38.9)
25–29 years	454 (30.8)	151 (29.2)	303 (31.7)
> =30 years	226 (15.4)	74 (14.3)	152 (15.9)
Education level			
Primary	488 (33.2)	185 (35.8)	303 (31.7)
Secondary	854 (58.0)	278 (53.8)	576 (60.3)
Tertiary	130 (8.8)	53 (10.4)	76 (8.0)
Marital status			
Unmarried	300 (20.4)	92 (17.8)	208 (21.8)
Married	1172 (79.6)	425 (82.2)	747 (78.2)
Wealth index			
Quintile1	489 (33.2)	184 (35.6)	305 (31.9)
Quintile2	100 (6.8)	31 (6.0)	69 (7.2)
Quintile3	298(20.2)	106 (20.5)	192 (20.1)
Quintile4	295 (20.0)	96 (18.6)	199 (20.8)
Quintile5	290 (19.7)	100 (19.3)	190 (19.9)
Gravidity			
First pregnancy	442 (30.0)	163 (31.5)	279 (29.2)
2–4 pregnancies	910 (61.8)	303 (58.6)	607 (63.6)
5 or more pregnancies	120 (8.2)	51 (9.9)	69 (7.2)
Hospitalisation during pregnancy			
No	1387 (94.2)	483 (93.4)	904 (94.7)
Yes	710 (94.5)	34 (6.6)	51 (5.3)
Antenatal visits			
Once	75 (5.1)	30 (5.8)	45 (4.7)
2–4 times	1259 (85.5)	442 (85.5)	817 (85.6)
5 or more times	138 (9.4)	45 (8.7)	93 (9.7)
Own domestic animals			
No	1355 (92.1)	472 (91.3)	883 (92.5)
Yes	117 (8.0)	45 (8.7)	72 (7.5)
Monthly income level			
< 30	205 (13.9)	76 (14.70)	129 (13.5)
30- < 60	587 (39.9)	216 (41.8)	371 (38.9)
60- < 90	454 (30.8)	151 (29.2)	303 (31.7)
≥ 90	226 (15.4)	74 (14.3)	152 (15.9)
Mode of delivery			
Spontaneous vaginal delivery	1334 (90.6)	480 (92.8)	854 (89.4)
Assisted vaginal delivery	135 (9.2)	35 (6.8)	100 (10.5)
Caesarean section	3 (0.2)	2 (0.4)	1 (0.1)
Sexual partners			
No	824 (70.3)	302 (71.1)	522 (69.9)
Yes	185 (15.8)	58 (13.7)	127 (17.0)
DNK	163 (13.9)	65 (15.3)	98 (13.1)
Premature rapture of membranes			
No	1466 (99.6)	512 (99.0)	954 (99.9)
Yes	6(0.4)	5 (1.0)	1 (0.1)
Prolonged labour			
No	1458 (99.1)	514 (99.4)	944 (98.9)
Yes	14 (0.9)	3 (0.6)	11 (1.1)
Use of mama kit			
No	1011 (68.7)	343 (66.3)	668 (69.9)
Yes	461 (31.3)	174 (33.7)	287 (30.1)
Tetanus toxoid vaccination			
No	1314 (89.3)	454 (87.8)	860 (90.1)
Yes	158 (10.7)	63 (12.2)	95 (9.9)

### Data collection and consent

Trained research nurses obtained verbal consent to collect specimens from women in labour, and after giving birth, obtained written informed consent to allow for the use of the collected specimens and data. Socio-demographic and clinical data were collected by using structured electronic questionnaires on mobile phones based on the Open Data Kit (ODK) software [14]. Distribution of relevant characteristics we collected can be found listed in Table 1. These include exposures associated with vaginal colonisation of mother, including premature rupture of membranes (PROM), defined as breakage of membranes of the amniotic sac before labour onset [15], prolonged labour, defined as labour beyond 24 h, parity, maternal level of education, maternal age, hospitalisation during pregnancy, marital status, antenatal care attendance, possession of domestic animals in the household, having been pregnant multiple times (multigravidity) and socioeconomic data.

### Specimen collection and transportation

Trained midwives collected vaginal swab specimens from the women during labour, by using Regular Rayon sterile swabs pre-packed with Amies Agar Gel without Charcoal transport medium (Copan Diagnostics Inc., Murrieta, CA). The swab was first carefully inserted into the vagina about halfway between the introitus and cervix. This way, contamination from the cervical mucus was avoided. The swab was then gently pressed towards the vaginal walls and rotated to ensure that it was thoroughly coated. The midwives took caution when removing the swab to avoid contact with the skin and the anal area. The vaginal swabs were immediately stored in the Amies transport medium in a specimen transport cooler. The coolers were subsequently transported within 24 h to MBN Clinical Laboratories where the specimens immediately underwent microbiological analyses [16].

### Microbiological analyses

Primary inoculation of the vaginal swabs was done on 5% sheep blood agar (BioLab Zrt., Budapest, Hungary) and on MacConkey agar (BioLab Zrt.), followed by aerobic incubation between 35 °C–37 °C for 18–24 h. The blood agar plates were further incubated for a total of 72 h to allow development of slow growing bacterial colonies. From these plates, we picked and streaked one representative of each morphologically distinct colony onto new agar plates and used colonies from this sub-culture for further species identification and characterisation.

Bacterial species identification was performed by using conventional microbiological techniques.

Gram-positive bacterial identification: *Staphylococcus aureus* species was identified based on positive catalase, coagulase and DNase tests. Beta-haemolytic Streptococci were identified by having distinct colony characteristics, having transparent haemolytic zones around colonies grown on blood agar plates, being Gram stain positive and being negative in the catalase test. The beta-haemolytic colonies were further Lancefield grouped into different species/groups (*Streptococcus* A-D) using the Streptococcal Grouping Kit (Oxoid Ltd., Basingstoke, Hants, UK). *Enterococcus* species were identified by being positive in the bile esculin test [17].

Gram-negative bacteria identification: These were identified biochemically based on lactose fermentation, triple sugar iron agar, sulfur-indole-motility, citrate and urease tests [17].

Reference strains *S. aureus* ATCC 25923 for gram-positive bacteria, and *E. coli* ATCC 25922 and *P. aeruginosa* ATCC 27853 for gram-negative bacteria were regularly included in the identification pipeline to control the quality of the microbiological procedures

### Main outcome and exposure definitions

The study’s main outcome was vaginal colonisation with potentially pathogenic bacteria. Colonisation with potential pathogenic bacteria was defined as isolation of at least one of the following types of bacteria from the vaginal swab; *S. aureus, E. coli*, *K. pneumoniae,* group A *Streptococcus* (GAS)*,* group B *Streptococcus* (GBS), *Enterococcus* spp.*, Pseudomonas* spp.*, Enterobacter* spp*., Citrobacter* spp*., Proteus* spp*.* and/or *Acinetobacter* spp*.* These bacteria are known to cause infections in newborns. Other bacteria that were isolated which we considered to represent commensal strains since they rarely are found associated with newborn infections included *Candida* spp., *Micrococcus* spp., *Corynebacterium* spp., *Lactobacillus* spp., *Bacillus* spp., *Bukolderia* spp., *Serratia* spp. and coagulase-negative *Staphylococcus*. Such isolates were not included in the analyses.

In the statistical analyses, described below, we tested for associations between different exposures and 4 different outcomes, including colonization with any potential pathogen, with *E. coli*, with *S. aureus*, and with *K. pneumoniae*. In those analyses, we tested exposures that other studies have found to be associated with maternal colonization with potentially pathogenic bacteria [18–21], including: Maternal level of education, maternal age, hospitalisation during pregnancy, marital status, number of previous pregnancies, antenatal tetanus toxoid vaccination, number of antenatal care visits, hospitalisations during pregnancy, possession of domestic animals at home, and socioeconomic status. Socioeconomic status was represented by a wealth index variable which was generated by performing principal component analysis on data about household ownership of cupboards, radios, televisions, a mobile phone, refrigerator, motor cycle, car, ownership of a house and/or land, and presence of cemented walls, type of toilet, and three or more rooms in the house. Five quintiles of the wealth index variables were generated with the poorest belonging to quintile 1, and the least poor to quintile 5.

### Statistical analysis

The data were analysed by using STATA 15.0 (StataCorp LLC, College Station, TX, USA). To obtain an estimate of the overall vaginal colonisation prevalence, we divided the number of women who had a positive vaginal culture for one or more potential pathogens by the total number of enrolled women in the study. To explore the associations between the above-mentioned outcomes and exposures, we performed bivariable (unadjusted) and multivariable (adjusted) logistic regression analyses where we estimated odds ratios (OR) and 95% confidence intervals (CIs) for each exposure. For each tested model we used the *estat vif* command in STATA to ensure there was little potential multicollinearity between the independent variables in the model, as indicated by one or more variance inflation factor estimates of > 10. None of our models appeared to have potential multicollinearity issues.

## Results

We recruited a total of 1472 women including 545 (37.0%) from Kawaala Health Centre III, 524 (36%) from Kitebi Health Centre III and 403 (27%) from Mukono Health Centre IV. The characteristics of these women are listed in Table 1. All but 3 (0.2%) of the women had vaginal deliveries. The mean age of the participants was 24.6 (standard deviation 4.9) years, 1172 (80%) were married or cohabiting, 1295 (88%) earned less than 30 US dollars per month, 185 (15.8%) had other sexual partners and 488 (33%) had at least a primary education. Only 6 (0.41%) of the women experienced PROM and 14 (0.9%) prolonged labour.

### Vaginal colonisation

Of the 1472 recruited women, 955 (64.9%; 95% CI 62.4, 67.3%) were colonised with at least one potential bacterial pathogen. Of the 955 colonised women, 878 were colonised with one potentially pathogenic bacteria, 69 were colonised with two potential pathogens while the remaining three women were colonised with three potential bacterial pathogens (Table 2). A total of 1025 potentially pathogenic bacterial pathogens were isolated from the colonised women. Overall, the most frequently isolated potential bacterial pathogens were *E. coli* (*n* = 508; 34.5%), *K. pneumoniae* (*n* = 145; 9.9%) and *S. aureus* (*n* = 121; 8.2%). There were no major differences in proportions of women colonised by potentially pathogenic bacteria between the three study sites (Table 3).

**Table 2:** Number and percentage of women colonised with more than one potentially pathogenic bacterial isolates

Combination of bacteria isolated	*N* = 1472 (%)
*Escherichia coli* and *Klebsiella pneumoniae*	10 (0.7)
*Escherichia coli* and *Enterococcus* spp.	10 (0.7)
*Escherichia coli* and *Staphylococcus aureus*	9 (0.6)
*Escherichia coli* and *Enterobacter* spp.	6 (0.4)
*Escherichia coli* and *Citrobacter* spp.	5 (0.3)
*Klebsiella pneumoniae* and *Enterococcus* spp.	4 (0.3)
*Escherichia coli and Escherichia coli*	4 (0.3)
*Staphylococcus aureus* and *Staphylococcus aureus*	4 (0.3)
*Klebsiella pneumoniae* and *Staphylococcus aureus*	3 (0.2)
*Klebsiella pneumoniae* and *Citrobacter* spp.	3 (0.2)
*Escherichia coli* and *Klebsiella oxytoca*	2 (0.1)
*Klebsiella oxytoca* and *Citrobacter* spp.	2 (0.1)
Ten other combinations^a^	1 (0.1)

^a^Includes: K. pneumoniae and Pseudomonas spp.; Acinetobacter spp. and S. aureus; E. coli and Proteus mirabilis; Enterococcus spp. and Citrobacter spp.; E. coli and Acinetobacter spp.; E. coli and Pseudomonas spp.; K. pneumoniae and K. pneumoniae; E. coli, Citrobacter spp., and S. aureus; E. coli, Pseudomonas spp., and S. aureus; E. coli, Enterococcus spp., and S. aureus

**Table 3:** Distribution of bacterial isolates from study participants across the three study sites

Bacteria isolated	Kawaala HC III *n* = 545 (%)	Kitebi HC III *n* = 524 (%)	Mukono HC IV *n* = 403 (%)	Total
				n = 1472 (%)
*E. coli*	203 (37.2)	183 (34.9)	122 (30.3)	508 (34.5)
*K. pneumoniae*	50 (9.2)	48 (9.2)	47 (11.7)	145 (9.9)
*S. aureus*	49 9.0)	41 (7.8)	31 (7.7)	121 (8.2)
*Citrobacter* spp.	38 (7.0)	38 (7.0)	31 (7.7)	107 (7.3)
*Enterococcus* spp.	16 (2.9)	19 (3.6)	12 (3.0)	47 (3.2)
*Enterobacter* spp.	21 (3.9)	5 (0.9)	6 (1.5)	32 (2.2)
*Acinetobacter* spp.	10 (1.8)	10 (1.9)	12 (3.0)	32 (2.2)
*K. oxytoca*	8 (1.5)	4 (0.8)	11 (2.7)	23 (1.6)
Group B *Streptococcus*	0	3 (0.6)	0	3 (0.2)
Group A *Streptococcus*	2 (0.14)	1 (0.07)	0	3 (0.2)
*Pseudomonas* spp.	0	3 (0.6)	0	3 (0.2)
*Proteus mirabilis*	1 (0.18)	0	0	1 (0.07)

### Exposures associated with vaginal colonisation

In the statistical analyses to identify exposures that are potentially associated with colonization with different pathogens, we found that having ≥5 previous pregnancies (aOR 0.59; 95% CI 0.35, 0.97) and maternal age of ≥30 years (aOR 1.52; 95% CI 1.03, 2.23) were associated with vaginal colonisation of women in labour with any potentially pathogenic bacteria (Table 4). Focusing these analyses on the three most commonly isolated potential pathogenic bacteria, we found that maternal age of ≥30 years (aOR 2.17; 95% CI 1.17, 4.00) and a history of at least 5 previous pregnancies (aOR 0.33; 95% CI 0.12, 0.88) were associated with *S. aureus* vaginal colonisation (Table 5). We found that possession of domestic animals in a household (aOR 0.57; 95% CI 0.35, 0.92) could be associated with vaginal colonisation by E. coli (Table 6). We found no exposures significantly associated with colonisation by *E. coli* (Table 6) and *K. pneumoniae* (Table 7).

**Table 4:** Exposures associated with vaginal colonisation with any potentially pathogenic bacteria of women in labour at three study sites (*N* = 1472)

Characteristics	*N* = 1472 (%)	Colonisation	
		Unadjusted OR (95% CI)	Adjusted OR (95% CI)
Age (years)			
20–24	587 (39.9)	1	1
≤ 19	205 (13.9)	0.99 (0.71, 1.37)	0.97 (0.68, 1.38)
25–29	454 (30.8)	1.17 (0.90, 1.51)	1.20 (0.91, 1.58)
≥ 30	226 (15.4)	1.20 (0.86, 1.65)	1.52 (1.03, 2.23)
Education level			
Tertiary	130 (8.8)	1	1
No education	33 (2.2)	0.75 (0.35, 1.63)	0.88 (0.40, 1.94)
Primary	455 (30.9)	1.20 (0.81, 1.79)	1.38 (0.91, 2.10)
Secondary	854 (58.0)	1.47 (1.01, 2.15)	1.64 (1.11, 2.43)
Gravida			
Primigravida	442 (30.0)	1	1
2–4 pregnancies	910 (61.8)	1.17 (0.92, 1.48)	1.06 (0.80, 1.40)
≥ 5 pregnancies	120 (8.2)	0.79 (0.52, 1.19)	0.59 (0.35, 0.97)
Hospitalisation during pregnancy			
No	1387 (94.2)	1	–
Yes	710 (94.5)	0.80 (0.51, 1.25)	–
Antenatal attendance			
One time	75 (5.1)	1	–
2–4 times	1259 (85.5)	1.23 (0.77, 1.98)	–
5 or more times	138 (9.4)	1.38 (0.77, 2.47)	–
Domestic animals at home			
No	1355 (92.1)	1	1
Yes	117 (8.0)	0.86 (0.58, 1.26)	0.80 (0.51, 1.25)
Wealth index			
5th Quintile (least poor)	290 (19.7)	1	1
1st Quintile (poorest)	489 (33.2)	0.87 (0.64, 1.18)	0.81 (0.57, 1.14)
2nd Quintile	100 (6.8)	1.17 (0.72, 1.91)	1.09 (0.65, 1.83)
3rd Quintile	298 (20.0)	0.95 (0.68, 1.34)	0.87 (0.60, 1.27)
4th Quintile	295 (20.0)	1.09 (0.77, 1.54)	1.01 (0.70, 1.46)

**Table 5:** Exposures associated with vaginal *S. aureus* colonisation of women in labour at three study sites (*N* = 1472)

Characteristics	*N* = 1472 (%)	*S. aureus* colonisation	
		Unadjusted OR (95% CI)	Adjusted OR (95% CI)
Age (years)			
20–24	587 (39.9)	1	1
≤ 19	205 (13.9)	1.10 (0.60, 2.00)	0.90 (0.48, 1.69)
25–29	454 (30.8)	1.12 (0.70, 1.78)	1.34 (0.81, 2.19)
≥ 30	226 (15.4)	1.47 (0.86, 2.51)	2.17 (1.17, 4.00)
Education level			
Tertiary	130 (8.8)	1	1
No education	33 (2.2)	0.87 (0.18, 4.22)	1.04 (0.21, 5.24)
Primary	455 (30.9)	1.33 (0.63, 2.82)	1.61 (0.73, 3.53)
Secondary	854 (58.0)	1.11 (0.54, 2.28)	1.27 (0.61, 2.67)
Gravida			
Primigravida	442 (30.0)	1	1
2–4 pregnancies	910 (61.8)	0.87 (0.58, 1.32)	0.67 (0.41 1.10)
≥ 5 pregnancies	120 (8.2)	0.64 (0.28, 1.47)	0.33 (0.12, 0.88)
Hospitalisation during pregnancy			
No	1387 (94.2)	1	–
Yes	710 (94.5)	1.04 (0.47, 2.3)	–
Antenatal attendance			
One time	75 (5.1)	1	–
2–4 times	1259 (85.5)	0.96 (0.41, 2.27)	–
5 or more times	138 (9.4)	1.30 (0.48, 3.53)	–
Domestic animals at home			
No	1355 (92.1)	1	1
Yes	117 (8.0)	0.72 (0.33, 1.58)	0.78 (0.32, 1.87)
Wealth index			
5th Quintile (Least poor)	290 (19.7)	1	1
1st Quintile (Poorest)	489 (33.2)	1.27 (0.74, 2.18)	1.14 (0.63, 2.09)
2nd Quintile	100 (6.8)	0.96 (0.40, 2.34)	0.84 (0.33, 2.13)
3rd Quintile	298 (20.0)	1.07 (0.58, 1.98)	0.99 (0.51, 1.93)
4th Quintile	295 (20.0)	1.03 (0.55, 1.92)	0.96 (0.50, 1.84)

**Table 6:** Exposures associated with vaginal *E. coli* colonisation of women in labour at three study sites (*N* = 1472)

Characteristics	*N* = 1472 (%)	*E. coli colonisation*	
		Unadjusted OR (95% CI)	Adjusted OR (95% CI)
Age (years)			
20–24	587 (39.9)	1	1
≤ 19	205 (13.9)	1.18 (0.85, 1.65)	1.10 (0.77, 1.57)
25–29	454 (30.8)	1.04 (0.80, 1.35)	1.06 (0.80, 1.40)
≥ 30	226 (15.4)	1.06 (0.77, 1.46)	1.20 (0.82, 1.75)
Education level			
Tertiary	130 (8.8)	1	1
No education	33 (2.2)	0.88 (0.37, 2.05)	0.94 (0.39, 2.26)
Primary	455 (30.9)	1.10 (0.72, 1.68)	1.15 (0.74, 1.80)
Secondary	854 (58.0)	1.33 (0.89, 1.98)	1.37 (0.91, 2.07)
Gravida			
Primigravida	442 (30.0)	1	1
2–4 pregnancies	910 (61.8)	0.96 (0.76, 1.22)	0.99 (0.74, 1.32)
≥ 5 pregnancies	120 (8.2)	0.75 (0.49, 1.17)	0.75 (0.43, 1.29)
Hospitalisation during pregnancy			
No	1387 (94.2)	1	–
Yes	710 (94.5)	0.74 (0.46, 1.21)	–
Antenatal attendance			
One time	75 (5.1)	1	–
2–4 times	1259 (85.5)	1.14 (0.69, 1.88)	–
5 or more times	138 (9.4)	0.87 (0.47, 1.59)	–
Domestic animals at home			
No	1355 (92.1)	1	1
Yes	117 (8.0)	0.64 (0.42, 0.98)	0.57 (0.35, 0.92)
Wealth index			
5th Quintile (Least poor)	290 (19.7)	1	1
1st Quintile (Poorest)	489 (33.2)	0.93 (0.69, 1.26)	0.77 (0.55, 1.09)
2nd Quintile	100 (6.8)	1.57 (0.99, 2.49)	1.30 (0.80, 2.11)
3rd Quintile	298 (20.0)	0.81 (0.57, 1.14)	0.67 (0.46, 1.0)
4th Quintile	295 (20.0)	0.96 (0.68, 1.35)	0.83 (0.57, 1.19)

**Table 7:** Exposures associated with vaginal *K. pneumoniae* colonisation of women in labour at three study sites (*N* = 1472)

Characteristics	*N* = 1472 (%)	*K. pneumoniae* colonisation	
		Unadjusted OR (95% CI)	Adjusted OR (95% CI)
Age (years)			
20–24	587 (39.9)	1	1
≤ 19	205 (13.9)	1.12 (0.67, 1.89)	1.45 (0.83, 2.55)
25–29	454 (30.8)	1.07 (0.71, 1.61)	0.96 (0.63,1.48)
≥ 30	226 (15.4)	0.8 (0.46, 1.40)	0.81 (0.43,1.51)
Education level			
Tertiary	130 (8.8)	1	1
No education	33 (2.2)	0.77 (0.21, 2.82)	0.73 (0.19, 2.80)
Primary	455 (30.9)	0.70 (0.37, 1.31)	0.65 (0.33, 1.27)
Secondary	854 (58.0)	0.88 (0.49, 1.58)	0.83 (0.46,1.52)
Gravida			
Primigravida	442 (30.0)	1	1
2–4 pregnancies	910 (61.8)	1.28 (0.86, 1.90)	1.49 (0.93, 2.36)
≥ 5 pregnancies	120 (8.2)	0.76 (0.49, 1.67)	1.05 (0.41, 2.68)
Hospitalisation during pregnancy			
No	1387 (94.2)	1	–
Yes	710 (94.5)	1.40 (0.73, 2.7)	–
ANC attendance			
One time	75 (5.1)	1	–
2–4 times	1259 (85.5)	1.49 (0.59,3.76)	–
5 or more times	138 (9.4)	2. 10 (0.75,5.90)	–
Domestic animals at home			
No	1355 (92.1)	1	1
Yes	117 (8.0)	1.17 (0.64, 2.14)	1.52 (0.74, 3.12)
Wealth index			
5th Quintile (least poor)	290 (19.7)	1	1
1st Quintile (poorest)	489 (33.2)	1.04 (0.62, 1.76)	1.22 (0.66, 2.25)
2nd Quintile	100 (6.8)	0.83 (0.35, 2.00)	0.97 (0.38, 2.47)
3rd Quintile	298 (20.0)	1.57 (0.91, 2.70)	1.82 (0.98, 3.39)
4th Quintile	295 (20.0)	1.44 (0.83, 2.50)	1.61 (0.88, 2.95)

## Discussion

We studied the prevalence of different potentially pathogenic bacteria colonising the vagina of women in labour at three primary health care facilities in Central Uganda and evaluated the association between potential risk factors and colonisation with these bacteria.

Sixty-five percent (65%) of the study participants were colonised by at least one potential bacterial pathogen. The prevalence of women colonised with potential pathogens in our study was higher than that reported in a similar study in Bangladesh [22]. This differences in colonisation prevalence may be due to several reasons, including differences in ethnic and geographical settings, that our study women were colonised with a wider range of pathogen species and the small sample size in the Bangladesh study. *E. coli, K. pneumoniae* and *S. aureus* were the most commonly isolated species. We found that the prevalences of individual potentially pathogenic bacteria were similar to those reported in other studies— *E. coli* was the predominant potential pathogen with a proportion similar to a study in Iran [23]. The proportion of *K. pneumoniae* isolates we found is similar to that reported in Nigeria [24] and Bangladesh [22]. Another study reported a prevalence of *S. aureus* vaginal colonisation in pregnant women similar to ours [25]. The bacteria that colonise the vagina of women in labour play an important role in newborn health such as defining their early gut microbiota [26]. A recent study has demonstrated that maternal vaginal colonisation with *E. coli* or *S. aureus* is significantly associated with pathogens isolated from the blood of neonates with early-onset sepsis [27].

In our study, the prevalence of vaginal GBS colonisation was only 0.2%, which is lower than what similar studies have reported [28, 29]. The difference could be a result of the methodological differences between our study and the other studies. We did not use the Todd Hewitt medium for GBS isolation, and did not collect anal swabs in our study, which could potentially have underestimated the GBS prevalence. The difference could also result from the fact that we use culture-based techniques to detect GBS instead of the more sensitive PCR based methods. However, vaginal colonisation varies greatly across geographical settings and a systematic review of studies from 85 countries indicates that East Africa and southern Asia have the lowest prevalence of maternal vaginal GBS colonisation compared to other regions [30]. Generally, we observed that there were no major differences in proportions of women colonised by potentially pathogenic bacteria between the study sites. This is an important finding because it indicates that this was a well-conducted large study and its findings are generalizable.

We found that women 30 years or more of age appeared more likely to be vaginally colonised with any potentially pathogenic bacteria and particularly with *S. aureus* compared to women who were 20–24 years in our study. Similar observations have been made in other studies [19, 21], where they found that older women were more often colonised than younger women. Vaginal colonisation rates during pregnancy may be attributed to several factors such as gestational age, mother’s age and parity. The association we observed could possibly be due to the fact that the majority of the women aged ≤30 years in our study were multipara and multigravida. We also found that women who had had at least 5 previous pregnancies appeared less likely to be colonised with these organisms than primigravida women. In contrast, studies in Thailand [31], Trinidad [19] and India [32] found multigravida women were more often colonised than primigravida women. These differences are difficult to explain, and given the exploratory nature of these analyses, further studies would be needed to confirm these results.

More surprising was the finding that women living with domestic animals at home were less likely to be colonised by *E. coli* than those who did not live with animals. Normally, living with animals would be considered an important risk factor for infection with *E. coli* [33, 34]. Further studies would be needed to identify the underlying reasons for why these women appeared to be protected. Few women in our study experienced premature rupture of membranes (PROM), which is an important risk factor for neonatal infections [35]. The low prevalence of PROM among the participants in our study is probably a result of the pre-delivery screening that is being done at our three health facilities, where women who are considered to be at risk of experiencing PROM or other complications during delivery are early on referred to tertiary hospitals.

One of the limitations of this study is that we only enrolled HIV-1 negative women. Nevertheless, we are confident that these findings are generalisable to the majority of women in reproductive age in Uganda because 92% of women of reproductive age in Uganda are HIV-1 negative. Since we used traditional microbiological methods to identify the different potential pathogenic bacteria, our prevalence estimates are probably lower than they would have been if we instead had used molecular profiling methods, such as PCR, to detect colonization.

## Conclusion

We found that among HIV-1 negative women in labour at health facilities in Central Uganda, almost two-thirds had vaginal colonisation by potentially pathogenic bacteria, mainly *E. coli*, *K. pneumoniae*, and *S. aureus*. This is of concern since exposures to pathogenic bacteria during birth is likely to increase the risk of newborn infections. We have also identified exposures that appear to be associated with colonisation with these potentially pathogenic organisms. Further studies are needed to evaluate the virulence of the potential pathogens and the risk of neonatal infections associated with this colonisation.

## Data Availability

Datasets used for this study can be obtained through a reasonable request from the principal investigator of the chlorhexidine trial (VN) nankabirwav@gmail.com and the corresponding author.

## Abbreviations

CI: Confidence interval
CISMAC: Centre for intervention science in maternal and child health
GAS: Group A *Streptococcus*
GBS: Group B *Streptococcus*
HIV-1: Human immunodeficiency virus type 1
NORHED: Norwegian programme for capacity development in higher education and research for development
ODK: Open data kit
OR: Odds ratio
PCR: Polymerase chain reaction
PROM: Premature rupture of membranes
SD: Standard deviation

## References

[1] Larsen B, Monif GR (2001). Understanding the bacterial flora of the female genital tract. Clin Infect Dis.

[2] Galask RP, Larsen B, Ohm MJ (1976). Vaginal flora and its role in disease entities. Clin Obstet Gynecol.

[3] Larsen B, Galask RP (1982). Vaginal microbial flora: composition and influences of host physiology. Ann Intern Med.

[4] Carroll SG, Papaioannou S, Ntumazah IL, Philpott-Howard J, Nicolaides KH (1996). Lower genital tract swabs in the prediction of intrauterine infection in preterm prelabour rupture of the membranes. Br J Obstet Gynaecol.

[5] Backhed F, Roswall J, Peng Y, Feng Q, Jia H, Kovatcheva-Datchary P, Li Y, Xia Y, Xie H, Zhong H (2015). Dynamics and stabilization of the human gut microbiome during the first year of life. Cell Host Microbe.

[6] Chan GJ, Modak JK, Mahmud AA, Baqui AH, Black RE, Saha SK (2013). Maternal and neonatal colonization in Bangladesh: prevalences, etiologies and risk factors. J Perinatol.

[7] GBD 2016 Mortality collaborators. Global, regional, and national under-5 mortality, adult mortality, age-specific mortality, and life expectancy, 1970–2016: A systematic analysis for the global burden of disease study 2016. Lancet. 2017;390(10100):1084–150. 10.1016/S0140-6736(17)31833-0 PMC5605514 28919115

[8] The Alliance for Maternal and Newborn Health Improvement (AMANHI) mortality study group. Population-based rates, timing, and causes of maternal deaths, stillbirths, and neonatal deaths in south Asia and sub-Saharan Africa: a multi-country prospective cohort study. Lancet Glob Health. 2018;6(12):e1297–308. 10.1016/S2214-109X(18)30385-1 PMC6227247 30361107

[9] Witkin SS, Linhares IM, Giraldo P (2007). Bacterial flora of the female genital tract: function and immune regulation. Clin Obstet Gynaecol.

[10] Ayengar V, Madhulika VSN (1991). Neonatal sepsis due to vertical transmission from maternal genital tract. Indian J Pediatr.

[11] Russell NJ, Seale AC, O'Sullivan C, Le Doare K, Heath PT, Lawn JE, Bartlett L, Cutland C, Gravett M, Ip M (2017). Risk of Early-Onset Neonatal Group B Streptococcal Disease With Maternal Colonization Worldwide: Systematic Review and Meta-analyses. Clin Infect Dis.

[12] Uganda population-based HIV impact assessment [https://phia.icap.columbia.edu/]. Accessed 24 August 2019.

[13] Nankabirwa V, Tylleskar T, Tumuhamye J, Tumwine JK, Ndeezi G, Martines JC, Sommerfelt H (2017). Efficacy of umbilical cord cleansing with a single application of 4% chlorhexidine for the prevention of newborn infections in Uganda: study protocol for a randomized controlled trial. Trials.

[14] Open Data Kit: The standard for mobile data collection [https://opendatakit.org]. Accessed 3 December 2018.

[15] Fishel Bartal M, Sibai BM, Ilan H, Fried M, Rahav R, Alexandroni H, Schushan Eisan I, Hendler I. Trial of labor after cesarean (TOLAC) in women with premature rupture of membranes. J Matern Fetal Neonatal Med. 2019:1–7. 10.1080/14767058.2019.1566312. 10.1080/14767058.2019.1566312 30652525

[16] MBN Clinical laboratories [https://mbnlab.com], Accessed 16 March 2019.

[17] Winn W. AS, Janda W., Koneman E., Procop G., Schreckenberger P., Woods G. Koneman’s Color Atlas and Textbook of Diagnostic Microbiology**.**, 6th Edition, edn. New York.: Lippincott Williams and Wilkins, New York.; 2006.

[18] Lederer DJ, Bell SC, Branson RD, Chalmers JD, Marshall R, Maslove DM, Ost DE, Punjabi NM, Schatz M, Smyth AR (2019). Control of confounding and reporting of results in causal inference studies. Guidance for authors from editors of respiratory, sleep, and critical care journals. Ann Am Thorac Soc.

[19] Orrett FA (2003). Colonization with group B streptococci in pregnancy and outcome of infected neonates in Trinidad. Pediatr Int.

[20] Stokholm J, Schjorring S, Eskildsen CE, Pedersen L, Bischoff AL, Folsgaard N, Carson CG, Chawes BL, Bonnelykke K, Molgaard A (2014). Antibiotic use during pregnancy alters the commensal vaginal microbiota. Clin Microbiol Infect.

[21] Khan MA, Faiz A, Ashshi AM (2015). Maternal colonization of group B streptococcus: prevalence, associated factors and antimicrobial resistance. Ann Saudi Med.

[22] Chan GJ, Modak JK, Mahmud AA, Baqui AH, Black RE, Saha SK (2013). Maternal and neonatal colonization in Bangladesh: prevalences, hh and risk factors. J Perinatol.

[23] Javanian M, Rad ZA, Mojaveri MH, Shiadeh AG, Ebrahimpour S (2017). Maternal recto vaginal colonization in term and preterm deliveries. Electron Physician.

[24] Ekwempu CC, Lawande RV, Egler LJ (1981). Microbial flora of the lower genital tract of women in labour in Zaria, Nigeria. J Clin Pathol.

[25] Andrews William W., Schelonka Robert, Waites Ken, Stamm Alan, Cliver Suzanne P., Moser Stephen (2008). Genital Tract Methicillin-Resistant Staphylococcus aureus. Obstetrics & Gynecology.

[26] Gabriel I, Olejek A, Stencel-Gabriel K, Wielgos M (2018). The influence of maternal vaginal flora on the intestinal colonization in newborns and 3-month-old infants. J Matern Fetal Neonatal Med.

[27] Kim JY, Sung JH, Chang KH, Choi SJ, Oh SY, Roh CR, Kim JH (2017). Abnormal vaginal colonization by gram-negative bacteria is significantly higher in pregnancy conceived through infertility treatment compared to natural pregnancy. J Matern Fetal Neonatal Med.

[28] Namugongo A, Bazira J, Fajardot Y, Joseph N (2016). Group B Streptococcus colonization among pregnant women attending antenatal Care at Tertiary Hospital in rural southwestern Uganda. Int J Microbiol.

[29] Ngonzi J, Bebell LM, Bazira J, Fajardo Y, Nyehangane D, Boum Y, Nanjebe D, Boatin A, Kabakyenga J, Jacquemyn Y (2018). Risk factors for vaginal colonization and relationship between bacterial vaginal colonization and in-hospital outcomes in women with obstructed labor in a Ugandan regional referral hospital. Int J Microbiol.

[30] Russell NJ, Seale AC, O'Driscoll M, O'Sullivan C, Bianchi-Jassir F, Gonzalez-Guarin J, Lawn JE, Baker CJ, Bartlett L, Cutland C (2017). Maternal Colonization With Group B Streptococcus and Serotype Distribution Worldwide: Systematic Review and Meta-analyses. Clin Infect Dis.

[31] Akkaneesermsaeng W, Petpichetchian C, Yingkachorn M, Sasithorn S. Prevalence and risk factors of group B Streptococcus colonisation in intrapartum women: a cross-sectional study. J Obstet Gynaecol. 2019:1–5. 10.1080/01443615.2019.1587597 31195907

[32] Sharmila V, Joseph NM, Arun Babu T, Chaturvedula L, Sistla S (2011). Genital tract group B streptococcal colonization in pregnant women: a south Indian perspective. J Infect Dev Ctries.

[33] Osman KM, Badr J, Orabi A, Elbehiry A, Saad A, Ibrahim MDS, Hanafy MH (2019). Poultry as a vector for emerging multidrug resistant Enterococcus spp.: first report of vancomycin (van) and the chloramphenicol-florfenicol (cat-fex-cfr) resistance genes from pigeon and duck faeces. Microb Pathog.

[34] Lupindu AM, Dalsgaard A, Msoffe PL, Ngowi HA, Mtambo MM, Olsen JE (2015). Transmission of antibiotic-resistant Escherichia coli between cattle, humans and the environment in peri-urban livestock keeping communities in Morogoro, Tanzania. Prev Vet Med.

[35] Ocviyanti D, Wahono WT (2018). Risk factors for neonatal Sepsis in pregnant women with premature rupture of the membrane. J Pregnancy.

